# Brain 3T magnetic resonance imaging in neonates: features and incidental findings from a research cohort enriched for preterm birth

**DOI:** 10.1136/archdischild-2024-326960

**Published:** 2024-07-02

**Authors:** Gemma Sullivan, Alan J Quigley, Samantha Choi, Rory Teed, Manuel Blesa Cabez, Kadi Vaher, Amy Corrigan, David Q Stoye, Michael J Thrippleton, Mark Bastin, James P Boardman

**Affiliations:** 1 The University of Edinburgh Centre for Clinical Brain Sciences, Edinburgh, UK; 2 Radiology, Royal Hospital for Children and Young People, Edinburgh, UK; 3 The University of Edinburgh MRC Centre for Reproductive Health, Edinburgh, UK; 4 The University of Edinburgh Edinburgh Imaging Facility, Edinburgh, UK

**Keywords:** magnetic resonance imaging, neonatology, neurology

## Abstract

**Background and objectives:**

The survival rate and patterns of brain injury after very preterm birth are evolving with changes in clinical practices. Additionally, incidental findings can present legal, ethical and practical considerations. Here, we report MRI features and incidental findings from a large, contemporary research cohort of very preterm infants and term controls.

**Methods:**

288 infants had 3T MRI at term-equivalent age: 187 infants born <32 weeks without major parenchymal lesions, and 101 term-born controls. T1-weighted, T2-weighted and susceptibility-weighted imaging were used to classify white and grey matter injury according to a structured system, and incidental findings described.

**Results:**

*Preterm infants*: 34 (18%) had white matter injury and 4 (2%) had grey matter injury. 51 (27%) infants had evidence of intracranial haemorrhage and 34 (18%) had punctate white matter lesions (PWMLs). Incidental findings were detected in 12 (6%) preterm infants. *Term infants*: no term infants had white or grey matter injury. Incidental findings were detected in 35 (35%); these included intracranial haemorrhage in 22 (22%), periventricular pseudocysts in 5 (5%) and PWMLs in 4 (4%) infants. From the whole cohort, 10 (3%) infants required referral to specialist services.

**Conclusions:**

One-fifth of very preterm infants without major parenchymal lesions have white or grey matter abnormalities at term-equivalent age. Incidental findings are seen in 6% of preterm and 35% of term infants. Overall, 3% of infants undergoing MRI for research require follow-up due to incidental findings. These data should help inform consent procedures for research and assist service planning for centres using 3T neonatal brain MRI for clinical purposes.

**WHAT IS ALREADY KNOWN ON THIS TOPIC**

- Neonatal MRI at term-equivalent age has established a common signature of preterm birth that includes diffuse white matter disease, reduced cortical grey matter complexity and enlarged cerebrospinal fluid spaces.
- All neuroimaging has the potential to detect incidental findings.
- There are intercohort variations in the reported prevalence of brain imaging features associated with preterm birth and incidental findings in the neonatal population.

**WHAT THIS STUDY ADDS**

- The prevalence of features of encephalopathy of prematurity identified on 3T brain MRI in a contemporaneous UK cohort without major parenchymal lesions imaged at term-equivalent age.
- The types and prevalence of incidental findings identified on 3T brain MRI in very preterm infants and term-born controls.

**HOW THIS STUDY MIGHT AFFECT RESEARCH, PRACTICE OR POLICY**

- This study will support clinicians and researchers with informed consent processes, image interpretation and service development.

## Introduction

 Advances in perinatal optimisation and neonatal intensive care have led to improved survival in infants born preterm.^1 2^ However, the risk of neurodisability in survivors remains high and this is largely due to preterm brain dysmaturation.^3^ While cerebral palsy affects <10% of very preterm infants, many more develop neurocognitive impairment and behavioural, social and emotional difficulties with pervasive effects on academic performance and lifecourse outcomes.^4^ Major focal injuries, including cystic periventricular leukomalacia (cPVL) are becoming less common, affecting <5% of very preterm infants.^5 6^ However, conventional neonatal MRI has established a common signature of preterm birth that includes diffuse white matter disease, enlargement of cerebrospinal fluid compartments and reduced cortical grey matter complexity. Structured scoring systems have been developed to grade these features.^7 8^ However, there are intercohort variations in the reported prevalence of these and other features associated with prematurity.^5 9^ There is a need to study a contemporaneous preterm cohort to identify the prevalence of these imaging features in the current era of neuroprotective strategies and modern-day intensive care practices.

Computational neonatal MRI has led to step-changes in mapping atypical patterns of brain development,^10^,^12^ and has advanced research into harmful exposures,^13^,^16^ neuroprotective strategies,^17^ prognostic tools^7 18 19^ and therapies.^20^,^23^ However, all imaging studies detect incidental findings, defined as ‘a finding that has potential health importance, unknown to the participant, which is discovered unexpectedly in the course of conducting research, but is unrelated to the purpose and beyond the aims of the study’.^24^ Incidental findings are common on brain MRI, with meta-analyses reporting a prevalence of 16%–22% in healthy children.^25 26^ Previous studies of term-born infants scanned soon after birth have reported an overall prevalence of incidental findings between 7% and 47%.^27^,^29^ Incidental findings are reported to be lower in preterm infants,^30 31^ although studies are few and case definition and acquisition protocols vary.

Knowledge of atypical brain features identified on neonatal brain MRI is essential for clinicians and researchers to support consent processes, guide interpretation of images and plan services. We used a 3T scanner to acquire T1-weighted (T1w), T2-weighted (T2w) and susceptibility-weighted imaging (SWI) in a neonatal research cohort. Study aims were: (1) to describe brain injury patterns in very preterm infants at term-equivalent age and (2) to describe the type and frequency of incidental findings in very preterm infants and term-born controls, and their impact on subsequent care pathways.

## Methods

### Participants

Theirworld Edinburgh Birth Cohort is a longitudinal cohort study of preterm infants (delivered before 33 weeks’ gestation) and healthy term-born controls.^32^ All cases were in-born. Infants who did not meet the following exclusion criteria were offered MRI at term-equivalent age: major congenital malformation, chromosomal abnormality, congenital infection, major parenchymal brain injury apparent on routine cranial ultrasound (cPVL, haemorrhagic parenchymal infarction, porencephalic cyst or posthaemorrhagic ventricular dilatation) and those with contraindications to MRI. Infants were scanned between December 2017 and December 2021. Preterm infants received cranial ultrasound scans using the anterior fontanelle window as part of routine care on days 1, 2, 3 and 7, and then weekly until 32 weeks, with a final scan between 36 and 40 weeks. Term infants had no neuroimaging prior to research MRI.

### MRI acquisition

A Siemens MAGNETOM Prisma 3T MRI scanner (Siemens Healthcare Erlangen, Germany) and 16-channel phased-array paediatric head and neck coil were used to acquire: three-dimensional (3D) T1w magnetisation-prepared rapid acquisition with gradient echo structural volume scan (voxel size=1 mm isotropic) with inversion time (TI) 1100 ms, echo time (TE) 4.69 ms, repetition time (TR) 1970 ms, flip angle 9°; a 3D T2w Sampling Perfection with Application-optimised Contrasts by using flip angle Evolution structural scan (voxel size=1 mm isotropic) with TE 409 ms and TR 3200 ms; a two-dimensional (2D) T2w BLADE (slice thickness=3 mm) with TE 207 ms and TR 4100 ms; axial 3D susceptibility-weighted imaging (TE=20 ms, TR=28 ms, flip angle 9°, 0.75×0.75×3.0 mm acquired resolution) and axial 2D fluid attenuated inversion-recovery BLADE imaging (TI=2606 ms, TE=130 ms, TR=10 000 ms, 0.94×0.94×3.0 mm acquired resolution).^32^

Infants were scanned at term-equivalent age, in natural sleep with monitoring of pulse oximetry, electrocardiography and temperature. Flexible ear plugs and earmuffs were used for acoustic protection. All scans were supervised by a doctor or nurse trained in neonatal resuscitation.

### MRI reporting

White matter and grey matter abnormalities were scored using a structured system.^8^ The white matter injury score was obtained by adding subscores of signal abnormality, periventricular volume loss, cystic abnormalities, ventricular dilatation and thinning of the corpus callosum. The grey matter injury score was obtained by adding subscores of cortical abnormalities, quality of gyral maturation and size of subarachnoid space. Each subscore was evaluated using a 3-point scale (1=normal, 2=mild abnormality, 3=moderate-to-severe abnormality). The categories of white matter injury were: normal (5–6), mild (7–9), moderate (10–12) or severe (13–15). Grey matter was categorised as normal if the score was <5, and abnormal if the score was 5–9. All MRI scans were reported by a paediatric radiologist (AJQ), and 50 randomly selected scans were reported by a second paediatric radiologist (SC). Inter-rater reliability was 95% in preterm infants, and there was complete agreement that the term scans showed no evidence of white or grey matter injury.

Incidental findings were defined according to the Royal College of Radiologists definition for research.^24^ Findings with diagnostic uncertainty or known clinical significance were discussed by a multidisciplinary team including specialists from neurosurgery, neurology, neuroradiology and clinical genetics to determine further investigations/imaging and follow-up.

Parents were counselled about the brain MRI results by a senior clinical member of the research team.

### Statistical analyses

For demographic features of study participants, two-sample t-tests were used to compare the means of normally distributed continuous variables, Wilcoxon rank-sum tests were applied to compare medians of non-normally distributed continuous variables and Fisher’s exact test was used to test for differences in categorical variables.

### Data availability

Requests for anonymised data will be considered under the study Data Access and Collaboration Policy (https://www.ed.ac.uk/centre-reproductive-health/tebc/about-tebc/for-researchers/data-access-collaboration).

## Results

### Participant characteristics

Of 291 infants who were eligible for MRI and whose parents consented, 3 (1%) were too unsettled to complete the imaging protocol. Data were acquired for 288 infants: 187 preterm (mean gestational age (GA) 29^+3^ weeks, range 22^+1^ to 32^+6^) and 101 term-born controls (mean GA 39^+4^ weeks, range 36^+3^ to 42^+1^). MRI scans were performed at 36^+2^ to 46^+1^ weeks. Term infants were imaged from 3 days to 6 weeks after birth (mean 17 days). 97% had T1w imaging, 99% had T2w imaging and 77% had SWI performed. Clinical and demographic features of participants are detailed in table 1. More infants in the preterm group were from the most deprived quintiles of the Scottish Index of Multiple Deprivation (p<0.001) and were delivered by caesarean section (p<0.004) when compared with term-born participants. Term-born infants were scanned at a greater postmenstrual age: mean 42^+0^ vs 40^+6^ weeks (p<0.001). The proportion of males and females was similar between groups (p=0.620).

**Table 1:** Participant characteristics

	Preterm, n=187	Term, n=101
Mean GA at birth, weeks (range)	29^+3^ (22^+1^–32^+6^)	39^+4^ (36^+3^–42^+1^)
Mean birth weight, g (SD)	1302 (409)	3495 (468)
Proportion of male infants (%)	105 (56%)	53 (53%)
Median birth weight *z-*score (range)	0.11 (−5.07–2.14)	0.51 (−2.30–2.57)
Mode of delivery, number (%)		
Vaginal	58 (31%)	36 (36%)
Vaginal assisted	3 (2%)	15 (15%)
Caesarean emergency	126 (67%)	18 (18%)
Caesarean elective	0 (0%)	32 (32%)
Scottish Index of Multiple DeprivationQuintile, number (%)		
1	35 (19%)	5 (5%)
2	34 (18%)	13 (12%)
3	33 (18%)	17 (17%)
4	37 (20%)	22 (22%)
5	48 (25%)	44 (44%)
Mean GA at MRI scan, weeks (range)	40^+6^ (36^+2^–45^+6^)	42^+0^ (38^+2^–46^+1^)
Antenatal steroid exposure, any (%)	179 (96%)	NA
Antenatal magnesium sulphate, any (%)	149 (80%)	NA
Bronchopulmonary dysplasia (%)	46 (25%)	NA
Early onset sepsis (%)	15 (8%)	NA
Late-onset sepsis (%)	30 (16%)	NA
Necrotising enterocolitis (%)	8 (4%)	NA
Retinopathy of prematurity (%)	9 (5%)	NA

GAgestational ageNAnot applicable

### MRI findings: preterm infants

Thirty-four (18%) preterm infants had an abnormal white matter injury score (>6); this was mildly abnormal in 30 (16%) infants and moderately abnormal in 4 (2%) infants. No preterm infants had severe white matter injury. Four (2%) preterm infants also had an abnormal grey matter injury score (online supplemental table 1).

In the subset of infants born before 28 weeks of gestation (n=49), 13 (27%) had an abnormal white matter injury score and 2 (4%) had an abnormal grey matter injury score.

The most common brain abnormalities were intracranial haemorrhage (ICH) and punctate white matter lesions (PWMLs) (table 2). Incidental findings were detected in 12 (6%) preterm infants.

**Table 2:** Summary of brain lesions and incidental findings

MRI findings	Prevalence
Preterm infants, n=85 (45%)	
Intracranial haemorrhage	51 (27%)
Intraventricular haemorrhage	48 (26%)
Cerebellar haemorrhage	10 (5%)
Subarachnoid haemorrhage	2 (1%)
Punctate white matter lesions	34 (18%)
Periventricular pseudocysts	18 (10%)
Connatal cysts	3 (2%)
Subependymal cysts	15 (8%)
Cystic white matter injury	2 (1%)
Suspected developmental venous anomaly	3 (2%)
Arachnoid cyst	1 (0.5%)
Middle cranial fossa cyst	1 (0.5%)
Cavum velum interpositum	1 (0.5%)
Subdural collection following meningitis	1 (0.5%)
Unusually shaped choroid plexus	1 (0.5%)
Giant cisterna magna	1 (0.5%)
Suspected pontine cerebellar hypoplasia	1 (0.5%)
Term infants, n=35 (35%)	
Intracranial haemorrhage	22 (22%)
Subdural	22 (22%)
Subarachnoid	1 (1%)
Germinal matrix haemorrhage	1 (1%)
Periventricular pseudocysts	5 (5%)
Connatal cysts	4 (4%)
Subependymal cysts	1 (1%)
Punctate white matter lesions	4 (4%)
Prominent extra-axial spaces	2 (2%)
Ventricular asymmetry	2 (2%)
Mild ventricular enlargement	1 (1%)
Fourth ventricle cyst	1 (1%)
Arachnoid cyst	1 (1%)
Developmental venous anomaly	1 (1%)
Low-lying cerebellar tonsils	1 (1%)
Subdural hygroma	1 (1%)

### Intracranial haemorrhage

**Figure 1:**
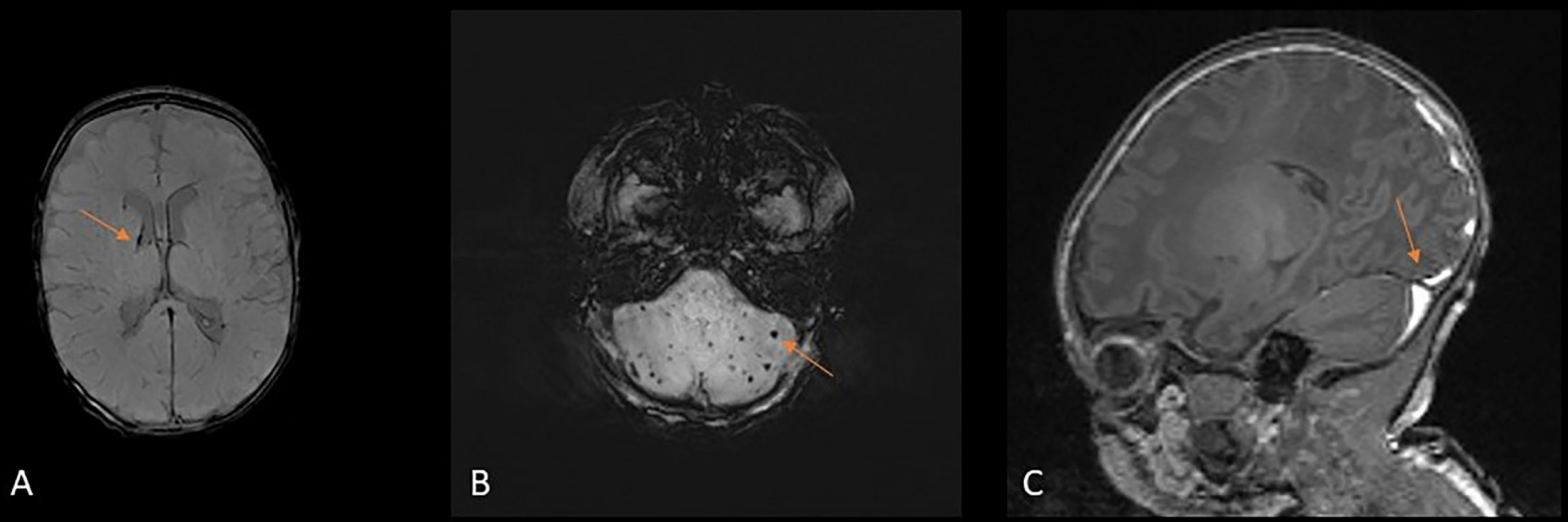
Intracranial haemorrhage. (**A**) susceptibility-weighted imaging (SWI) of infant aged 30^+3^ weeks, scanned at 40^+2^ weeks, showing residual germinal matrix haemorrhage-intraventricular haemorrhage. (**B**) SWI of infant aged 30^+0^ weeks, scanned at 40^+4^ weeks, showing multiple punctate cerebellar haemorrhages. (**C**) T1-weighted image of infant aged 39^+2^ weeks, scanned at 40^+3^ weeks, showing subdural haemorrhage.

Previous ICH was identified in 51 (27%) infants. Forty-eight (26%) infants had resolving germinal matrix haemorrhage-intraventricular haemorrhage (GMH-IVH), 10 (5%) had cerebellar haemorrhage and 2 (1%) had subarachnoid haemorrhage (SAH). Example images are shown in figure 1A-C.

### Punctate white matter lesions

**Figure 2:**
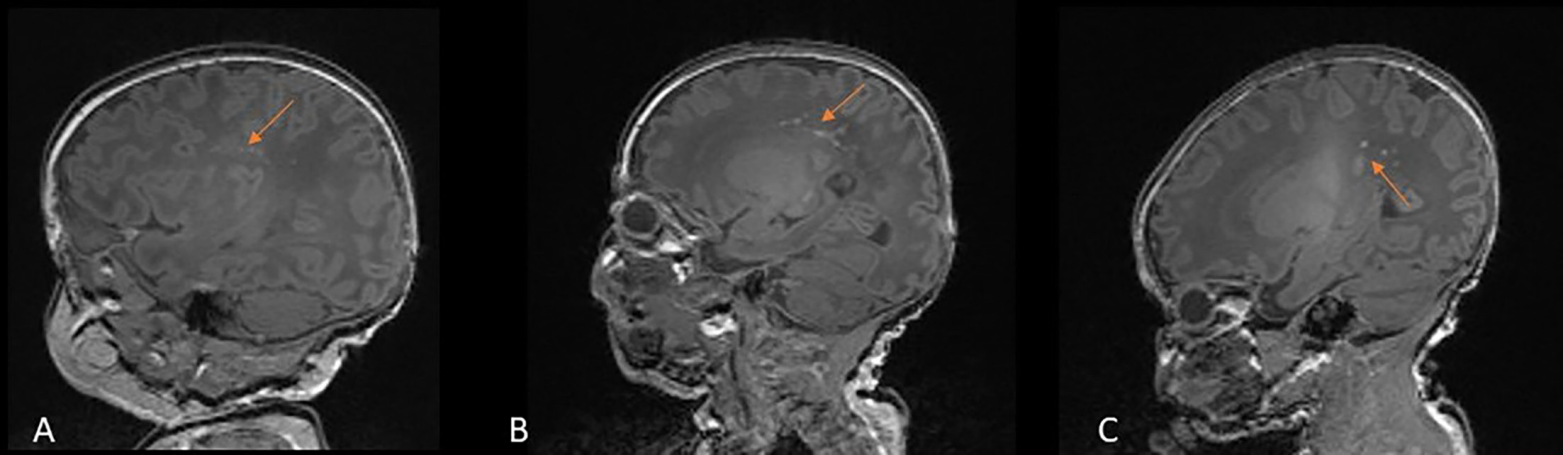
Punctate white matter lesions. T1-weighted images showing punctate white matter lesions in (**A**) infant aged 32^+0^ weeks, scanned at 44^+2^ weeks, (**B**) infant aged 31^+3^ weeks, scanned at 39^+0^ weeks, (**C**) infant aged 39^+1^ weeks, scanned at 40^+5^ weeks.

PWMLs with increased signal intensity on T1w imaging and decreased signal intensity on T2w imaging were seen in 34 (18%) infants (figure 2). Fourteen infants had <3 lesions and 20 infants had multiple lesions. Thirty-one infants with PWMLs had SWI performed. Twenty-six infants (84%) showed no loss of signal on SWI, suggesting non-haemorrhagic origin. Four infants had haemorrhagic PWMLs and one infant had a mixture of haemorrhagic and non-haemorrhagic lesions.

### Incidental findings

**Figure 3:**
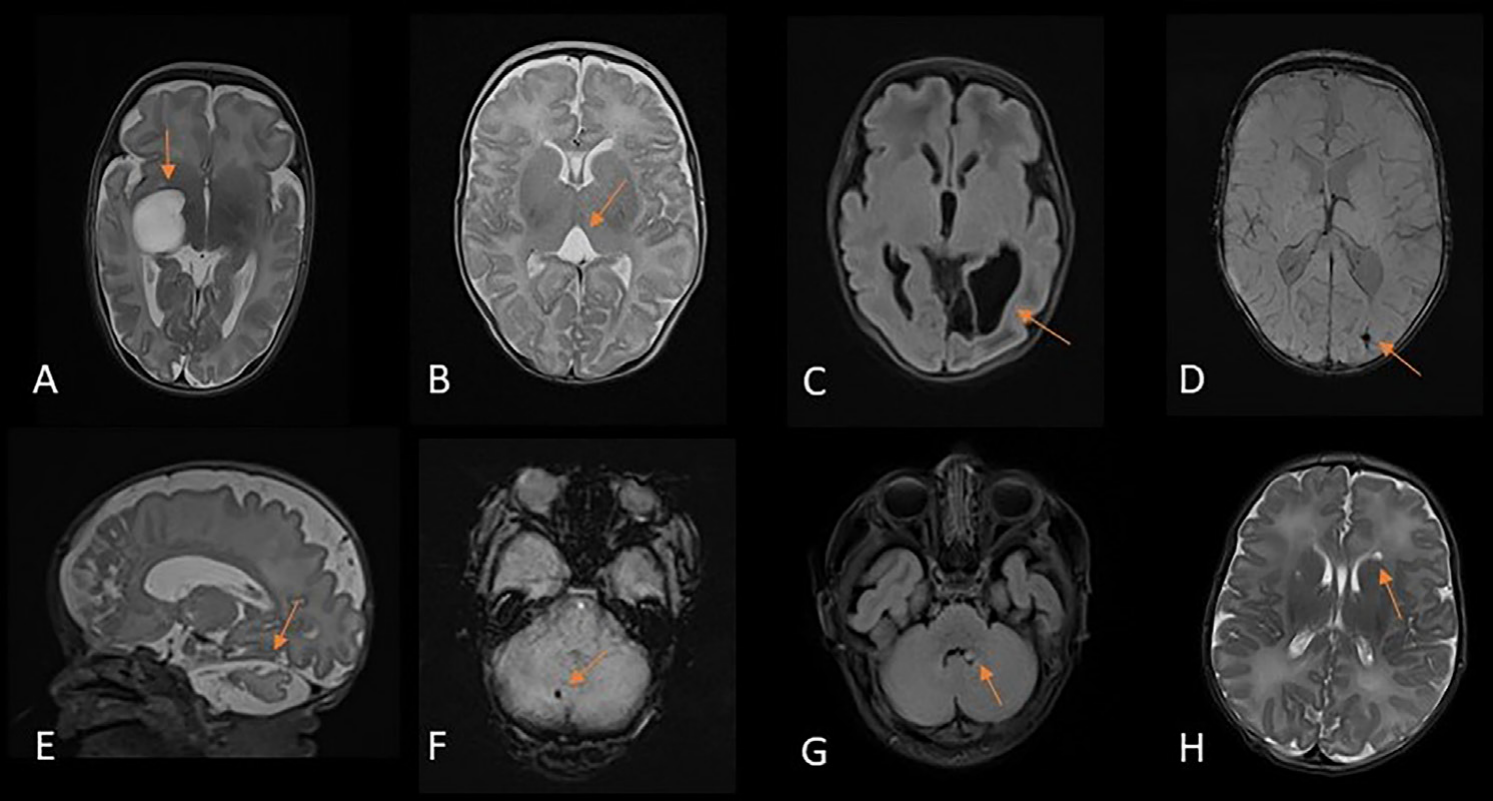
Incidental findings. (**A**) T2-weighted image of an infant aged 27^+4^ weeks, scanned at 42^+1^ weeks with a large middle cranial fossa cyst. (**B**) T2-weighted image of an infant aged 28^+5^ weeks, scanned at 40^+1^ weeks showing a cavum velum interpositum. (**C**) T2 BLADE of an infant aged 32^+3^ weeks, scanned at 36^+2^ weeks with cystic change in the left occipital lobe. (**D**) SWI of an infant aged 27^+2^ weeks, scanned at 42^+0^ weeks, demonstrating cystic change in the left occipital lobe. (**E**) T2 image of an infant aged 29^+3^ weeks, scanned at 40^+4^ weeks, demonstrating increased subarachnoid space and suspected pontine cerebellar hypoplasia. (**F**) SWI of an infant aged 31^+1^ weeks, scanned at 37^+6^ weeks, demonstrating a focus of abnormality between the right cerebellar hemisphere and vermis due to developmental venous anomaly. (**G**) T2 BLADE of an infant aged 40^+1^ weeks, scanned at 43^+3^ weeks, showing a 4 mm cyst on the floor of the fourth ventricle. (**H**) T2 BLADE of an infant aged 39^+1^ weeks, scanned at 43^+0^ weeks, showing a left connatal cyst.

Incidental findings were identified in 12 (6%) preterm infants (figure 3A-F). Two (1%) infants had white matter cysts identified on MRI that were not seen previously on routine cranial ultrasound (figure 3C,D). Developmental venous anomaly (DVA) was suspected for three infants on initial MRI. Repeat MRI confirmed the diagnosis of DVA for one infant while appearances were more suggestive of a linear PWML and a cerebellar haemorrhage for the other two infants, respectively. Other incidental findings included a middle cranial fossa cyst (figure 3A), giant cisterna magna, suspected pontine cerebellar hypoplasia and cavum velum interpositum (figure 3B).

### MRI findings: term infants

No term-born infants had white matter or grey matter injury that breached the abnormality threshold, although 7 (7%) had subthreshold scores in white matter and 11 (11%) in grey matter (online supplemental table 1). Incidental findings were detected in 35 (35%) term-born infants (table 2).

### Intracranial haemorrhage

ICH was seen in 22 (22%) infants. Twenty infants had isolated subdural haemorrhage (SDH). One infant had both SDH and GMH-IVH and one infant had both SDH and SAH. The majority of SDH were located in the posterior fossa, but nine infants had evidence of supratentorial haemorrhage (figure 1C).

Similar to previous reports,^27^ we found that infants with ICH were delivered vaginally or by emergency caesarean, with no haemorrhage identified in any infant born by elective caesarean section (n=32). SDH was identified in 31% of infants born by spontaneous vaginal delivery and 47% of infants born by assisted vaginal delivery.

### Punctate white matter lesions

PWMLs with increased signal intensity on T1w imaging and decreased signal intensity on T2w imaging were identified in four (4%) infants (figure 2). Two infants had <3 lesions and two had multiple lesions. None demonstrated signal change on SWI, suggesting an ischaemic origin.

### Other incidental findings

Other incidental findings were identified in 15 infants, including cysts (figure 3G-H), low-lying cerebellar tonsils, subdural hygroma and DVA (table 2).

### Outcomes for infants with incidental findings

Overall, 10 (8 (4%) preterm and 2 (2%) term) infants were referred to clinical services and eight required further neuroimaging, detailed in online supplemental table 2.

## Discussion

We report 3T brain MRI findings from a large research cohort of very preterm infants and term-born controls.

Among preterm infants, previous GMH-IVH was the most common finding, affecting 48 (26%) infants. This is higher than previous estimates (14%–19%)^5 33^ and may be because we included blood-sensitive SWI sequences in our study. Thirty-four (18%) preterm infants had mild-to-moderate global white matter injury scores and four (2%) also had an abnormal grey matter score. These prevalences are slightly lower than those described by Leuchter *et al*, who reported abnormal white matter scores in 22%–36% and abnormal grey matter scores in 7%–19%, and other classification schemes.^7 8 34^ The differences are most likely explained by variation in study populations because we excluded infants with major parenchymal lesions identified on routine cranial ultrasound. It is also possible that differences in perinatal neuroprotection exposures, classification of diffuse white matter signal intensity and different feature detection rates between 3T and 1.5T MRI scanners may contribute. Inter-rater agreement for categorising white and grey matter injuries was high, and adds further support for the use of structured reporting tools in neonatal MRI studies.

PWMLs are common in preterm infants and lesion load has been associated with increased risk of poor motor outcomes.^35^ PWMLs in term-born infants have been associated with perinatal hypoxia ischaemia^36^ and congenital heart disease,^37^ but they are also observed in healthy controls. PWMLs were identified in 34 (18%) of our preterm cohort and four (4%) term-born controls. A recent systematic review reported a 22% incidence of PWMLs in preterm infants scanned at term-equivalent age.^35^ PWMLs may represent venous infarction with haemorrhage or increased cellularity due to gliosis. The inclusion of blood-sensitive SWI sequences in this study enabled distinction between haemorrhagic and non-haemorrhagic PWMLs.

In term infants, SDH was the most common incidental finding, affecting 22 (22%) neonates. This aligns with data from cohorts with similar rates of instrumental delivery, imaged within the first 2 weeks of life.^27^ While the incidence was higher in infants delivered with assistance (47%) compared with spontaneous vaginal delivery (31%), our data also showed SDH in infants born by emergency caesarean section (22%). Reassuringly, longitudinal data show no association between SDH in term-born infants and abnormal neurodevelopmental outcome.^27^

Overall, we found a high rate of incidental findings. Although the majority were not serious, others have identified arterial ischaemic stroke, ectopic posterior pituitary, spinal cord compression and tuberous sclerosis in the neonatal period.^5 30 31^

A strength of this study is the large sample size of prospectively recruited preterm infants and contemporaneous term-born controls. Images were obtained using the same 3T brain-optimised research MRI scanner using a standardised protocol. All scans were reviewed by a paediatric radiologist using a structured system with excellent inter-rater agreement and prognostic value. Potential limitations of this study regarding generalisability of results include the use of 3D isotropic T1w and T2w imaging, which may be more sensitive to findings, and the single-centre study population. However, the very preterm cohort is representative of a typical UK population with respect to fetal neuroprotection, and neonatal morbidity. Term-born controls were recruited from a variety of healthcare settings to enable comparison of different modes of delivery and facilitate representation from all five quintiles of the Scottish Index of Multiple Deprivation. A potential limitation is that we scanned the preterm infants at term-equivalent age, which may not capture the dynamic changes in injury patterns reported in studies that include multiple data acquisitions over time.^38 39^ Finally, for preterm infants the incidental findings definition relies on prior knowledge from routine ultrasound, so may be subject to variation across settings due to factors such as frequency of scanning, hardware and the windows used for image acquisition.

## Conclusion

One-fifth of very preterm infants without major parenchymal injuries have white or grey matter abnormalities apparent on 3T MRI at term-equivalent age. Incidental findings are common in both preterm infants and term-born controls but the majority are not clinically actionable. Overall, 3% of infants require referral to specialist services for additional investigation. Given that incidental findings can present legal, ethical and practical considerations that impact individuals and health services, these data will support the informed consent process, assist image interpretation and inform the development of guidelines for managing incidental findings detected in neonatal neuroimaging studies.

## supplementary material

10.1136/archdischild-2024-326960 online supplemental file 1

## Data Availability

Data are available on reasonable request.

## References

[1] Cao G, Liu J, Liu M (2022). Global, regional, and national incidence and mortality of neonatal preterm birth, 1990-2019. JAMA Pediatr.

[2] Smith LK, van Blankenstein E, Fox G (2023). Effect of national guidance on survival for babies born at 22 weeks' gestation in England and Wales: population based cohort study. *BMJ Med*.

[3] Inder TE, Volpe JJ, Anderson PJ (2023). Defining the neurologic consequences of preterm birth. N Engl J Med.

[4] Johnson S, Marlow N (2017). Early and long-term outcome of infants born extremely preterm. Arch Dis Child.

[5] Arulkumaran S, Tusor N, Chew A (2020). MRI findings at term-corrected age and neurodevelopmental outcomes in a large cohort of very preterm infants. *AJNR Am J Neuroradiol*.

[6] Bell EF, Hintz SR, Hansen NI (2022). Mortality, in-hospital morbidity, care practices, and 2-year outcomes for extremely preterm infants in the US, 2013-2018. JAMA.

[7] Woodward LJ, Anderson PJ, Austin NC (2006). Neonatal MRI to predict neurodevelopmental outcomes in preterm infants. N Engl J Med.

[8] Leuchter RH-V, Gui L, Poncet A (2014). Association between early administration of high-dose erythropoietin in preterm infants and brain MRI abnormality at term-equivalent age. JAMA.

[9] Guillot M, Sebastianski M, Lemyre B (2021). Comparative performance of head ultrasound and MRI in detecting preterm brain injury and predicting outcomes: a systematic review. Acta Paediatrica.

[10] Galdi P, Blesa M, Stoye DQ (2020). Neonatal morphometric similarity mapping for predicting brain age and characterizing neuroanatomic variation associated with preterm birth. Neuroimage Clin.

[11] Blesa M, Galdi P, Cox SR (2020). Hierarchical complexity of the macro-scale neonatal brain. Cerebral Cortex.

[12] Dimitrova R, Pietsch M, Ciarrusta J (2021). Preterm birth alters the development of cortical microstructure and morphology at term-equivalent age. Neuroimage.

[13] Stoye DQ, Blesa M, Sullivan G (2020). Maternal cortisol is associated with neonatal amygdala microstructure and connectivity in a sexually dimorphic manner. Elife.

[14] Sullivan G, Galdi P, Cabez MB (2020). Interleukin-8 dysregulation is implicated in brain dysmaturation following preterm birth. Brain Behav Immun.

[15] Mckinnon K, Galdi P, Blesa-Cábez M (2023). Association of preterm birth and socioeconomic status with neonatal brain structure. JAMA Netw Open.

[16] Duerden EG, Grunau RE, Guo T (2018). Early procedural pain is associated with regionally-specific alterations in thalamic development in preterm neonates. *J Neurosci*.

[17] Sullivan G, Vaher K, Blesa M (2023). Breast milk exposure is associated with cortical maturation in preterm infants. Ann Neurol.

[18] van Kooij BJ, de Vries LS, Ball G (2012). Neonatal tract-based spatial statistics findings and outcome in preterm infants. AJNR Am J Neuroradiol.

[19] Lally PJ, Montaldo P, Oliveira V (2019). Magnetic resonance spectroscopy assessment of brain injury after moderate hypothermia in neonatal encephalopathy: a prospective multicentre cohort study. Lancet Neurol.

[20] Poppe T, Thompson B, Boardman JP (2022). Effect of antenatal magnesium sulphate on MRI biomarkers of white matter development at term equivalent age: the magnum study. EBioMedicine.

[21] Wu YW, Comstock BA, Gonzalez FF (2022). Trial of erythropoietin for hypoxic–ischemic encephalopathy in newborns. N Engl J Med.

[22] Alison M, Tilea B, Toumazi A (2020). Prophylactic hydrocortisone in extremely preterm infants and brain MRI abnormality. Arch Dis Child Fetal Neonatal Ed.

[23] Moltu SJ, Nordvik T, Rossholt ME (2024). Arachidonic and docosahexaenoic acid supplementation and brain maturation in preterm infants; a double blind RCT. Clin Nutr.

[24] The Royal College of Radiologists(RCR) (2011). Management of incidental findings detected during research imaging.

[25] Dangouloff-Ros V, Roux C-J, Boulouis G (2019). Incidental brain MRI findings in children: a systematic review and meta-analysis. AJNR Am J Neuroradiol.

[26] Li Y, Thompson WK, Reuter C (2021). Rates of incidental findings in brain magnetic resonance imaging in children. JAMA Neurol.

[27] Carney O, Hughes E, Tusor N (2021). Incidental findings on brain MR imaging of asymptomatic term neonates in the developing human connectome project. EClinicalMedicine.

[28] Kumpulainen V, Lehtola SJ, Tuulari JJ (2019). Prevalence and risk factors of incidental findings in brain Mris of healthy neonates—the Finnbrain birth cohort study. Front Neurol.

[29] Looney CB, Smith JK, Merck LH (2007). Intracranial hemorrhage in asymptomatic neonates: prevalence on MR images and relationship to obstetric and neonatal risk factors. Radiology.

[30] Malova M, Rossi A, Severino M (2017). Incidental findings on routine brain MRI scans in preterm infants. Arch Dis Child Fetal Neonatal Ed.

[31] Buchiboyina A, Yip CSA, Madhala S (2019). Incidental findings on brain magnetic resonance imaging in preterm infants. Neonatology.

[32] Boardman JP, Hall J, Thrippleton MJ (2020). Impact of preterm birth on brain development and long-term outcome: protocol for a cohort study in Scotland. BMJ Open.

[33] Kidokoro H, Anderson PJ, Doyle LW (2014). Brain injury and altered brain growth in preterm infants: predictors and prognosis. Pediatrics.

[34] Kidokoro H, Neil JJ, Inder TE (2013). New MR imaging assessment tool to define brain abnormalities in very preterm infants at term. AJNR Am J Neuroradiol.

[35] de Bruijn CAM, Di Michele S, Tataranno ML (2023). Neurodevelopmental consequences of preterm punctate white matter lesions: a systematic review. Pediatr Res.

[36] Hayman M, van Wezel-Meijler G, van Straaten H (2019). Punctate white-matter lesions in the full-term newborn: underlying aetiology and outcome. Eur J Paediatr Neurol.

[37] Guo T, Chau V, Peyvandi S (2019). White matter injury in term neonates with congenital heart diseases: topology & comparison with preterm newborns. Neuroimage.

[38] Kersbergen KJ, Benders MJNL, Groenendaal F (2014). Different patterns of punctate white matter lesions in serially scanned preterm infants. PLoS One.

[39] Martinez-Biarge M, Groenendaal F, Kersbergen KJ (2016). MRI based preterm white matter injury classification: the importance of sequential imaging in determining severity of injury. PLoS One.

