# Supplementary material for: Brain 3T magnetic resonance imaging in neonates: features and incidental findings from a research cohort enriched for preterm birth
Source: Arch Dis Child Fetal Neonatal Ed. 2024 Jul 2;110(1):e326960. doi: 10.1136/archdischild-2024-326960 (PMC11672019; doi:10.1136/archdischild-2024-326960)
Supplement: online supplemental file 1 [file fetalneonatal-110-1-s001.pdf]

Supplementary Table 1. Numerical brain injury scores

	Number (%) with Abnormal Score	
**Outcomes**	**Preterm, n=187**	**Term, n=101**
White matter injury (score 7 or more)	34 (18%)	0 (0%)
Sub-scores of white matter injury (score 2 or 3)		
White matter signal abnormality	56 (30%)	3 (3%)
Periventricular white matter loss	19 (10%)	0 (0%)
Cystic abnormalities	5 (3%)	0 (0%)
Ventricular dilatation	52 (28%)	4 (4%)
Thinning of corpus callosum	25 (13%)	0 (0%)
Gray matter injury (score 5 or more)	4 (2%)	0 (0%)
Sub-scores of gray matter injury (score 2 or 3)		
Cortical signal abnormality	2 (1%)	1 (1%)
Quality of gyral maturation	3 (2%)	0 (0%)
Subarachnoid space	151 (81%)	10 (10%)

An abnormal white matter injury score is defined as 7 or more on a scale of 5 to 15, and an abnormal gray matter score is defined as 5 or more on a scale of 3 to 9. An abnormal sub-score is defined as 2 or 3 on a scale of 1 to 3.

Supplementary Table 2. Outcomes for infants referred to clinical services

Incidental finding	Outcome
**Preterm infants, n=8**	
Possible developmental venous anomaly right corona radiata	Repeat MRI at 3 months showed no vascular abnormality. A small area of bright FLAIR signal remained, likely secondary to periventricular white matter injury.
Possible developmental venous anomaly left cerebellar dentate nucleus	Repeat MRI 1 month later showed no vascular abnormality and appearances were suggestive of previous cerebellar haemorrhage.
Possible developmental venous anomaly right cerebellar hemisphere.	Repeat MRI 1 month later showed an unchanged focus of SWI signal abnormality in the right cerebellar hemisphere, likely confirmation of a developmental venous anomaly.
Well defined 0.9 x 1.1 x 1.8 cm cyst superior to the cerebral veins in the region of the pineal gland/quadrigeminal cistern with CSF signal on all sequences. Differential diagnosis included cavum velum interpositum, arachnoid cyst or pineal cyst.	Repeat MRI including constructive interference in steady state (CISS) volumetric sequence 2 weeks later showed no change in size. Probable cavum septum interpositum.
Large CSF-filled cystic area in right middle cranial fossa measuring 2.5 x 2.6 x 2.5cm in size and exerting a mass effect on the temporal lobe and midbrain.	Cystic area thought to represent an encysted temporal horn/choroidal fissure. Referred to Neurosurgery but no intervention was required.
Evidence of cystic change in the left occipital lobe. Prominent CSF space inferior to the cerebellar vermis, raises the possibility of a giant cisterna magna or arachnoid cyst.	Repeat MRI 1 month later showed similar appearances of the left occipital lobe. The prominent CSF space inferior to the cerebellar vermis was consistent with a giant cisterna magna.
Unusual globular appearance of the posterior aspect of the left choroid plexus within the left ventricular trigone.	Repeat MRI 6 weeks later still showed a prominent left choroid plexus, but it was smaller and thought to be of no clinical significance.
Deep grey matter calcification and suspected pontine cerebellar hypoplasia.	Referred to Neurology and Clinical Genetics but no diagnosis made. Developed complex neurodisability and died following withdrawal of life sustaining treatment at 8 months of age.
**Term infants, n=2**	
4mm cyst in the fourth ventricle with high T2/low T1 signal suggesting fluid content.	Repeat MRI 1 month later with constructive interference in steady state (CISS) volumetric sequence showed the lesion persisted but had not changed in size. Differential diagnosis included neuroepithelial cyst, colloid cyst or neuroenteric cyst. Follow-up MRI was recommended but parents declined.

Well-defined extra-axial collection extending from lateral aspect of right frontal lobe to anterior pole of right temporal lobe inferiorly. There are no vascular markings and appearances suggest a subdural hygroma.	Repeat MRI 1 month later showed a smaller than previous subdural collection, presumed secondary to delivery.
